# Eye Movement Evidence for Hierarchy Effects on Memory Representation of Discourses

**DOI:** 10.1371/journal.pone.0147313

**Published:** 2016-01-20

**Authors:** Yingying Wu, Xiaohong Yang, Yufang Yang

**Affiliations:** 1 Key Laboratory of Behavioral Science, Institute of Psychology, Chinese Academy of Sciences, Beijing, China; 2 University of Chinese Academy of Sciences, Beijing, China; University of Leicester, UNITED KINGDOM

## Abstract

In this study, we applied the text-change paradigm to investigate whether and how discourse hierarchy affected the memory representation of a discourse. Three kinds of three-sentence discourses were constructed. In the hierarchy-high condition and the hierarchy-low condition, the three sentences of the discourses were hierarchically organized and the last sentence of each discourse was located at the high level and the low level of the discourse hierarchy, respectively. In the linear condition, the three sentences of the discourses were linearly organized. Critical words were always located at the last sentence of the discourses. These discourses were successively presented twice and the critical words were changed to semantically related words in the second presentation. The results showed that during the early processing stage, the critical words were read for longer times when they were changed in the hierarchy-high and the linear conditions, but not in the hierarchy-low condition. During the late processing stage, the changed-critical words were again found to induce longer reading times only when they were in the hierarchy-high condition. These results suggest that words in a discourse have better memory representation when they are located at the higher rather than at the lower level of the discourse hierarchy. Global discourse hierarchy is established as an important factor in constructing the mental representation of a discourse.

## Introduction

Human language is represented in the brain in hierarchical ways [1], and the processing of hierarchical structures is a core feature that differentiates human language from other animal communication systems e.g., [2, 3]. Previous studies have found that processing different levels of hierarchy may involve different cognitive and neural mechanisms during sentence comprehension e.g., [4, 5, 6]. It is therefore of great interest to investigate whether and how hierarchy affects cognitive processing, such as the construction of mental representations during discourse comprehension.

Studies at the sentence level have repeatedly shown that the hierarchical organization of a sentence plays an important role in sentence comprehension. First, hierarchy affects online syntactic analysis. Compared with subordinate clauses, syntactic error in main clauses induces smaller P600 effect with earlier latency, indicating that syntactic analysis in main clauses was less difficult than that in subordinate clauses [7]. Second, hierarchy interacts with inter-clause semantic relation and clause order, affecting the availability of information. In particular, information in main clauses is more accessible than that in subordinate clauses, especially when the clauses are linked by temporal conjunction, e.g., *after* [8–10]. Third, hierarchy affects the processing depth of information. For example, Baker and Wagner [6] found that false information was more likely to be reported when it was conveyed by main clauses than by subordinate clauses. This result was replicated by another experiment with text-change paradigm [5]. In addition to the studies of complex sentences, Jiang and Zhou [4] investigated neural dynamics in processing different levels of syntactic hierarchy during simple sentence processing. The results showed that violating the low-level constraints elicited a left-lateralized, anteriorly maximized negativity, whereas violating the high-level constraints elicited a right anterior negativity (RAN) and a right centro-parietal negativity (N400), suggesting that different levels of syntactic hierarchy may involve different neural mechanisms.

Equally important, but has received far less attention, is the effect of hierarchy on a larger language unit, i.e., discourse. According to Rhetorical Structure Theory (RST), coherence of a discourse is supported by a hierarchical, connected structure of the discourse [11, 12]. Two sentences in a discourse that share the same intended function are joined to create a span and to convey the purpose of the writer. This span may, in turn, be related to another sentence or span to create a new effect on the reader. This process continues until the end of a discourse, resulting in a hierarchical structure of the discourse. Discourse hierarchy can affect the depth of semantic integration during comprehension. Discourse-incongruous words were read longer than discourse-congruous words only when the critical sentence and the preceding sentence were located at the same discourse level, but not when they were located at different discourse levels [13]. In addition, studies of prosody have shown that people tend to insert longer preceding pauses for clauses located at the high level than for those located at the low level [14, 15], which indicates that the representation of discourse hierarchy is kept in mind during the planning of speech production. Despite these studies, whether and how hierarchy affects cognitive mechanisms during discourse processing, such as the construction of mental representations, remains unresolved.

To address this issue, we constructed three-sentence discourses with various structures according to RST. In the hierarchy-high and hierarchy-low conditions, the three sentences of the discourses were hierarchically organized and the last sentence of each discourse was located at the high level and the low level of the discourse hierarchy respectively. In the linear condition, the three sentences of the discourses were organized linearly. The stimuli in Table 1 are examples.

**Table 1:** Experimental stimuli of the present study.

Condition	Discourses
Hierarchy-low condition	严立/想为/写作课/取材/, 他/去/房间/拿/放大镜/, 蹲下/观察/**蚱蜢/ (蜈蚣) / **生活/习性/。
	Yanli/wanted to/writing class/collected source material/, he/went/room/took/magnifying glass/, squatted/observed/ **grasshoppers/ (centipedes)**/living/habits/.
	Yanli wanted to collect source material for the writing class. He took a magnifying glass from his room, and squatted to observe the living habits of the **grasshoppers (centipedes)**.
Hierarchy-high condition	严立/洒了/一点/食物/, 从/房间/拿来/放大镜/, 他/打算/观察/**蚱蜢/ (蜈蚣) / **生活/习性/。
	Yanli/dropped/some/food/, from/room/took/magnifying glass/, he/wanted to/observed/ **grasshoppers /(centipedes)**/living/habits/.
	Yanli dropped some food on the floor, and took a magnifying glass from his room. He wanted to observe the living habits of the **grasshoppers (centipedes)**.
Linear condition	严立/放/食物/后/起身/, 去/房间/拿来/放大镜/, 再/蹲下/观察/**蚱蜢/ (蜈蚣) / **生活/习性/。
	Yanli/dropped/food/after/stood up/, went/room/took/magnifying glass/, then/squatted/observed/ **grasshoppers/ (centipedes)**/living/habits/.
	Yanli dropped some food on the floor and stood up. He took a magnifying glass from his room. He squatted to observe the living habits of the **grasshoppers (centipedes)**.

*Note*. Critical words are marked in bold. The changed-critical words are presented in parentheses.

**Fig 1:**
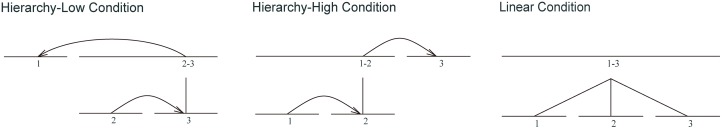
Hierarchical structures for example discourses.

In the hierarchy-low condition, the first sentence briefly describes the goal of Yanli, and both the second and the third sentences elaborate what Yanli did to achieve this goal. The latter two sentences are closely related to each other and share the same intention. In terms of their effects on readers, they should be joined into a span before they are connected to the first sentence. According to RST, these two elaboration sentences occupy the lower levels of the discourse hierarchy, and the first goal sentence occupies the higher level of the discourse hierarchy, as shown in Fig 1. In the hierarchy-high condition, the previous two sentences specifically depict the actions of Yanli, whereas the last sentence describes the purpose of Yanli’s actions. Therefore, the previous two sentences are sub-units for the same discourse intention, and they create a span first before they are related to the last sentence. In this case, the final sentence is located at the higher level of the discourse hierarchy (central in Fig 1). In the linear condition, all the three sentences describe the actions of Yanli, such that all of them share the common discourse intention and are located at the same level of the discourse hierarchy (right in Fig 1). The critical word *grasshoppers* is always located in the last sentence of the discourses. These discourses are successively presented twice with an interposing blank screen, and the critical word is changed to the semantically related word *centipedes* on the second presentation.

Previous studies have shown that higher-level information receives deeper processing during sentence comprehension [5, 6] and that humans are sensitive to hierarchical structure during discourse processing [13]. Therefore, we hypothesize that hierarchy will modulate the memory representation of the discourses, such that the change of the critical words in the hierarchy-high condition will be detected most successfully, whereas the change of those critical words in the hierarchy-low condition will be detected least successfully.

## Method

### Ethics Statement

The experiment was approved by the Institutional Review Board of the Institute of Psychology, Chinese Academy of Sciences. The participants signed informed consent forms before the experiment and had the right to quit the experiment at any time.

### Participants

Thirty undergraduate students (16 women; mean age = 21.0 years, *SD* = 1.9) participated in the experiment. All were native Chinese speakers with normal or corrected-to-normal vision. All were paid for participation.

### Apparatus

Eye movements were monitored using EyeLink 1000, which had a sampling rate of 1000 Hz. The participants viewed the stimuli with both eyes; however, only the right eye was recorded. Stimuli were presented in 28 Chinese Songti font on a 21-inch CRT monitor with a refresh rate of 150 Hz and a resolution of 1024×768 pixels. Participants read the discourses approximately 58 cm from the monitor.

### Materials

Thirty-six sets of three-sentence discourses were constructed (see Table 1 for an example). Sentences in a discourse were connected by commas to ensure that the effects of syntactic markings would not confound the effects of discourse structure [13]. The critical words (all two-character words) appeared in the middle of the final sentences, followed by two two-character words. The words that immediately preceded and followed the critical words were identical across all the three conditions. The critical words in the two displays were matched on stroke number [first display, *M* = 17.72, *SD* = 4.07; second display, *M* = 17.94, *SD* = 3.75] and log frequency [first display, *M* = 3.24, *SD* = .83; second display, *M* = 3.31, *SD* = .86]; all *t*s<−.72. The semantic relatedness of the critical words between the two displays was 3.26 (.22) on a scale ranging from 1 (*no relevance*) to 5 (*very strong relevance*), indicating moderate relatedness of critical words between the two displays.

Three offline pretests of the experimental materials were conducted to assess the structures, the predictability of the critical words, and the coherence of the discourses. In the structure pretest, 24 participants were asked to choose one structure for each discourse from the three structures as shown in Fig 1. The results showed that the approval rates for the discourse structure were high, and there was no difference among the three structures, *F*<1 (as shown in Table 2). In the cloze probability pretest, another 24 participants were asked to complete the discourses that were truncated before the critical words. The results revealed that the probabilities for predicting the critical words on both displays were low, and there was no difference among the three conditions, *F*_S_<1. In the coherence pretest, another 24 participants were asked to rate the coherence of the discourses on a scale of 1 (*not coherent at all*) to 5 (*very coherent*). The results showed that there was no difference across the conditions and displays, *F*_S_<1.

**Table 2:** Pretests of experimental materials.

	Discourse structure	Cloze probability	Discourse coherence
**First display**			
*Hierarchy-low condition*	99.31% (4.17%)	0(0)	3.52(0.70)
*Hierarchy-high condition*	99.65% (2.08%)	0(0)	3.52(0.70)
*Linear condition*	98.61% (3.98%)	0.35%(2.08%)	3.47(0.63)
**Second display**			
*Hierarchy-low condition*	-	0.35%(2.08%)	3.51(0.66)
*Hierarchy-high condition*	-	0(0)	3.54(0.56)
*Linear condition*	-	0.35%(2.08%)	3.66(0.61)

*Note*. Stander deviations are presented in parentheses.

The experimental discourses were divided into three versions via Latin square design. Each version contained 36 discourses (12 per condition). Besides, 108 filler discourses were added to the experimental materials. A total of 36 fillers matched the experimental items in discourse structures, with 12 discourses per structure. These fillers remained unchanged on the second presentation. The 72 other fillers had different discourse structures from the experimental materials. For half of these fillers, words changed at a variety of locations. For the other half of these fillers, words remained identical. In total, the participants read 144 discourses during the experimental phase, half of which included changes. There were 8 practice discourses before each experimental session.

### Procedure

Participants were instructed that each discourse would be presented twice, and their task was to read for comprehension. In addition, they were asked to read at their normal pace and read through each discourse only once if they already understood the discourse [16, 17].

A calibration was conducted at the beginning of the experiment. Each trial started with a drift calibration, after which a small square appeared at the position where the first character of upcoming discourses would be located. The participants were asked to fixate on the square until it disappeared and was replaced by a discourse. The discourses were presented as a whole, with each sentence in one separate line. The participants were asked to press a handheld button after they finished reading the discourses. Once they pressed the button, the discourses disappeared and were replaced by a blank screen for 500 ms. The second display of the discourses then appeared on the screen. For 1/3 of the trials, the participants were asked to answer a comprehension question by pressing marked buttons. The entire experiment lasted for approximately 60 minutes.

### Data analysis

Data obtained from one female subject were eliminated from the data analysis because the trials in which reading paths were chaotic or in which she blinked were over one-third of her total trials. The average accuracy rate of the 29 other subjects was 90.03%. Further 6.9% of the remaining trials were excluded because of the fixation disruptions or blinks. Fixations less than 80 ms were merged into the nearest fixation point if they were less than one character at a distance. Other fixations less than 80 ms or more than 800 ms were omitted. This procedure excluded a total of 1.22% fixation points. Finally, reading times that were three standard deviations above or below the mean for the critical words of a given subject in a given condition were deleted, which removed 0.07% of the total data.

Four eye movement measures were examined: first fixation duration (the duration of the first first-pass fixation on a region), gaze duration (the time a reader spends on a region before the reader leaves it), go-pass time (the sum of the time spent on a region and to the left of the region before moving to the right of it), and total time (the sum of all fixation durations on a region). The first two measures were associated with early processing, and the last two measures were associated with later processing [18, 19]. We did not count the instances of skipping for the first fixation duration, gaze duration, go-pass time, and total time; thus, 10.34%, 10.60%, 10.70%, and 4.58%, respectively, of the total data were removed from final analysis.

For each eye movement measure, a 3 (hierarchy: hierarchy-high, hierarchy-low, and linear) × 2 (text change: first-display, second-display) repeated ANOVA was conducted, with participants (*F*_1_) and items (*F*_2_) as random factors.

## Results

Table 3 shows the means and standard deviations for the critical words.

**Table 3:** Eye movement measures on the critical words.

	Hierarchy-low condition	Hierarchy-high condition	Linear condition
First fixation duration (ms)			
*first display*	258(86)	263(85)	274(104)
*second display*	275(107)	284(100)	284(98)
Gaze duration (ms)			
*first display*	381(216)	345(169)	357(185)
*second display*	378(231)	405(231)	400(231)
Go-pass time (ms)			
*first display*	481(343)	419(258)	435(278)
*second display*	445(282)	470(283)	452(293)
Total time (ms)			
*first display*	498(295)	453(246)	443(228)
*second display*	470(288)	509(279)	471(275)

*Note*. Standard deviations are presented in parentheses.

For the first fixation duration, the main effect of text change was found. The changed-critical words in the second display were read longer than the original-critical words in the first display, *F*_1_(1, 28) = 11.03, *MSE* = 981, *p* < .01, $\eta_{p}^{2}$ = .28; *F*_2_(1, 35) = 19.90, *MSE* = 711, *p* < .001, $\eta_{p}^{2}$ = .36. Neither a main effect of hierarchy nor an interaction was found, *F*_S_<2.15.

No main effect of hierarchy was found on gaze duration, *F*_S_<1. However, a main effect of text change was found; the changed-critical words were read longer than the original-critical words, *F*_1_(1, 28) = 7.65, *MSE* = 7013, *p* < .05, $\eta_{p}^{2}$ = .22; *F*_2_(1, 35) = 7.77, *MSE* = 6976, *p* < .01, $\eta_{p}^{2}$ = .18. More importantly, the text change effect was modulated by hierarchy, *F*_1_(2, 56) = 5.22, *MSE* = 3590, *p* < .01, $\eta_{p}^{2}$ = .16; *F*_2_(2, 70) = 4.94, *MSE* = 5530, *p* < .05, $\eta_{p}^{2}$ = .12. In the hierarchy-low condition, no significant difference was found between the two displays, *F*_S_<1. However, in the hierarchy-high condition, the gaze durations were longer for the changed-critical words than for the original-critical words, *F*_1_(1, 28) = 12.58, *MSE* = 4843, *p* < .01, $\eta_{p}^{2}$ = .31; *F*_2_(1, 35) = 13.58, *MSE* = 4523, *p* < .01, $\eta_{p}^{2}$ = .28. This was also the case for the linear condition, *F*_1_(1, 28) = 5.79, *MSE* = 5164, *p* < .05, $\eta_{p}^{2}$ = .17; *F*_2_(1, 35) = 5.51, *MSE* = 8063, *p* < .05, $\eta_{p}^{2}$ = .14.

For go-pass time, no significant differences were found among the three hierarchical levels or between the two displays, *F*_S_<1.20. However, an interaction effect was found between them, *F*_1_(2, 56) = 5.42, *MSE* = 5339, *p* < .01, $\eta_{p}^{2}$ = .16; *F*_2_(2, 70) = 4.28, *MSE* = 8884, *p* < .05, $\eta_{p}^{2}$ = .11. Simple effect analysis revealed that in the hierarchy-high condition, the changed-critical words were read longer than the original-critical words, *F*_1_(1, 28) = 4.99, *MSE* = 8853, *p* < .05, $\eta_{p}^{2}$ = .15; *F*_2_(1, 35) = 8.54, *MSE* = 5355, *p* < .01, $\eta_{p}^{2}$ = .20, whereas in the hierarchy-low condition or in the linear condition, the times spent on the two displays had no significant differences, *F*_S_<2.56.

The results for total time showed a similar pattern as the go-pass time. Neither the main effect of hierarchy nor the main effect of text change was significant, *F*_s_<2.05. However, the interaction was remarkable, *F*_1_(2, 56) = 6.47, *MSE* = 4928, *p* < .01, $\eta_{p}^{2}$ = .19; *F*_2_(2, 70) = 4.95, *MSE* = 6878, *p* < .05, $\eta_{p}^{2}$ = .12. In the hierarchy-high condition, the total times were longer in the second display than in the first display, *F*_1_(1, 28) = 6.47, *MSE* = 8610, *p* < .05, $\eta_{p}^{2}$ = .19; *F*_2_(1, 35) = 8.21, *MSE* = 5429, *p* < .01, $\eta_{p}^{2}$ = .19. However, no significant differences were found between the two displays in the hierarchy-low condition or in the linear condition, *F*_S_<2.19.

## Discussion

Our findings showed that during the early processing stage, the critical words were read longer when they were changed in the hierarchy-high and linear conditions, but not in the hierarchy-low condition. Moreover, during the late processing stage, the changed-critical words induced longer reading times, but only in the hierarchy-high condition.

The changed-critical words induced longer first fixation duration for each condition, indicating the same rapid and automatic processing of lexical access for the changed-critical words across all the three conditions [20, 21]. More importantly, longer gaze duration for the changed-critical words was observed in the hierarchy-high and linear condition, but not in the hierarchy-low condition. This result suggests that information at the higher level of the discourse hierarchy is represented in finer granularity and that readers are more sensitive to the change at the higher level than that at the lower level of the discourse hierarchy. Note that of the hierarchy-high and the linear condition, the changes in the hierarchy-high condition are more salient than those in the linear condition for readers. As evidenced by the fact that for the hierarchy-high condition, longer reading times were observed both on the early processing measure (gaze duration) and the late processing measures (go-pass time and total time), whereas for the linear condition, longer reading times were found only on the early processing measure. These results suggest that the memory representation of the original-critical words vary as a function of discourse hierarchy.

The finding that information at the higher level of discourse hierarchy is represented in finer granularity extends previous studies at phrase and sentence levels, which suggested that information at the higher level had a better memory representation than information at the lower level [5, 22]. In addition, our results compare favorably with the previous finding that central sentences of discourse had a better memory representation than sentences that were loosely related with text theme e.g., [23].

The moderate effect of discourse hierarchy on memory representation of a discourse may be related to the uneven prominence of different hierarchical levels. According to RST [11, 12], information at the higher level played a more prominent role in expressing a writer’s intention, which was supported by acoustic studies showing that speakers inserted a longer pause at boundaries of higher hierarchy [14, 15]. In studies using the change-detection paradigm, more successful detection of the changes was taken as an indication that more attention resources was allocated to the elements being changed e.g., [24, 25, 26]. Therefore, our finding that the changed-critical words in the hierarchy-high condition were more successfully detected could be due to the fact that information at the higher level of the discourse hierarchy received more attention and was processed to a deeper extent.

Another possibility may be related to the activation of propositions. In the framework of construction-integration theory for discourse comprehension, highly activated propositions would predicate a better memory representation [27]. Possibly, the information located at the higher level was more highly activated than that at the lower level. Therefore, the critical words in the hierarchy-high condition had a better memory representation than those in the linear or hierarchy-low condition.

Givón [28] argued that linguistic cues could function as mental processing instructions, instructing readers in the importance of concepts and how to allocate attention during discourse comprehension. This has been evidenced by numerous studies that suggested that linguistic cues as lexical devices and syntactic devices influenced the processing depth and memory representation of discourses e.g., [29, 30–32]. However, all of these studies focused on the linguistic cues related to lexical and syntactic devices at the sentence level. Thus, our findings contribute to the literature by showing that linguistic cues as signaled by hierarchical organization at the discourse level also play an important role in discourse comprehension.

In summary, the current study provides evidence that information has a better memory representation when it is located at the higher level than at the lower level of the discourse hierarchy, suggesting that information in a discourse is represented in various granularities as a function of hierarchical levels. Our results extend previous findings on hierarchy processing at the phrase and sentence levels and establish discourse hierarchy as an important linguistic device that modulates the memory representation of a discourse.
